# Supplementary material for: Signature of cardiac alterations in early and late chronic infections with Trypanosoma cruzi in mice
Source: PLoS One. 2023 Oct 5;18(10):e0292520. doi: 10.1371/journal.pone.0292520 (PMC10553825; doi:10.1371/journal.pone.0292520)
Supplement: S1 Table — (DOCX) [file pone.0292520.s001.docx]

**S1 Table. Reclassification matrix of individual mice according to LDA analysis of ECG patterns.**

|  |  | **Predicted group** | |  |  |  |
| --- | --- | --- | --- | --- | --- | --- |
| **Actual Balb/c** | Uninfected | Acute | Early Chronic | Late Chronic | Total | Correct |
| Uninfected | 12 | 8 | 5 | 9 | 34 | 35.3 % |
| Acute | 2 | 16 | 4 | 4 | 26 | 61.5% |
| Early Chronic | 3 | 8 | 11 | 4 | 26 | 42.3% |
| Late Chronic | 6 | 3 | 4 | 12 | 25 | 48.0% |
| Total |  |  |  |  |  | 45.9% |
| **Actual ICR** | Uninfected | Acute | Early Chronic | Late Chronic | Total | Correct |
| Uninfected | 26 | 1 | 4 | 3 | 34 | 76.5% |
| Acute | 5 | 2 | 5 | 3 | 15 | 13.3% |
| Early Chronic | 3 | 1 | 9 | 3 | 16 | 56.3% |
| Late Chronic | 0 | 1 | 3 | 12 | 16 | 75.0% |
| Total |  |  |  |  |  | 60.5% |

Balb/c and ICR mice from the actual groups indicated were reclassified based on the LDA analysis of their ECG patterns. Counts and correct percentage of reclassified individual mice is indicated for each group.
