# Supplementary material for: Signature of cardiac alterations in early and late chronic infections with Trypanosoma cruzi in mice
Source: PLoS One. 2023 Oct 5;18(10):e0292520. doi: 10.1371/journal.pone.0292520 (PMC10553825; doi:10.1371/journal.pone.0292520)
Supplement: S2 Table — (DOCX) [file pone.0292520.s002.docx]

**S2 Table.** **Reclassification matrix of individual mice according to LDA analysis of ECG patterns.**

|  |  | **Predicted group** | |  |  |  |
| --- | --- | --- | --- | --- | --- | --- |
| **Actual group** | Uninfected ICR | ICR Late Chronic | Uninfected Balb/c | Balb/c Late Chronic | Total | Correct |
| Uninfected ICR | 5 | 0 | 0 | 1 | 6 | 83.3 % |
| ICR Late Chronic | 1 | 7 | 0 | 0 | 8 | 87.5% |
| Uninfected Balb/c | 0 | 0 | 6 | 0 | 6 | 100% |
| Balb/c Late Chronic | 0 | 0 | 2 | 10 | 12 | 83.3% |
| Total |  |  |  |  | 32 | 87.5% |
