# Supplementary material for: Signature of cardiac alterations in early and late chronic infections with Trypanosoma cruzi in mice
Source: PLoS One. 2023 Oct 5;18(10):e0292520. doi: 10.1371/journal.pone.0292520 (PMC10553825; doi:10.1371/journal.pone.0292520)
Supplement: Attachment — Submitted filename: PONE-D-23-22437.pdf ccorrected.pdf [file pone.0292520.s003.pdf]

PLOS ONE

Signature of cardiac alterations in early and late chronic infections with Trypanosoma cruzi in mice

--Manuscript Draft--

**Manuscript Number:**	PONE-D-23-22437
**Article Type:**	Research Article
**Full Title:**	Signature of cardiac alterations in early and late chronic infections with Trypanosoma cruzi in mice
**Short Title:**	Cardiac alterations in Trypanosoma cruzi infected-mice
**Corresponding Author:**	Eric Dumonteil, Ph.D.<br>Tulane University School of Public Health and Tropical Medicine<br>New Orleans, LA UNITED STATES
**Keywords:**	Chagas disease, Trypanosoma cruzi, Electrocardiography, Echocardiography, Chronic infection.
**Abstract:**	Chagas disease by Trypanosoma cruzi (T. cruzi) infection is a leading cause of myocarditis worldwide. Chagas cardiomyopathy is presented with a wide variety of conduction abnormalities including arrhythmias, first- and second-degree atrioventricular blockade, left ventricular systolic dysfunction and some cases heart failure leading to the death. Currently, there are no effective treatments available against advanced Chagas disease. With the advance in the development of novel therapies, it is important to utilize an animal model that can effectively replicate the diverse stages of Chagas disease, including chronic asymptomatic and symptomatic infection, that are akin to those observed in humans. Therefore, to characterize the cardiac alterations during the evolution of the infection, we evaluated the progression of cardiomyopathy caused by T. cruzi H1 infection in both Balb/c and ICR mouse models by performing electrocardiogram (ECG) studies in unanesthetized mice every month until 210 days post-infection (dpi). In the late chronic phase of infection, we also performed echocardiogram (ECHO) studies to further assess cardiac function. We demonstrate that ICR mice were more susceptible to cardiac alterations compared to Balb/c mice and suitable as an experimental chronic infection model.
**Order of Authors:**	Barbara Carolina Arias Argaez
	Victor Manuel Dzul Huchim
	Ana Paulina Haro Álvarez
	Miguel Enrique Rosado Vallado
	Liliana Villanueva Lizama
	Julio Vladimir Cruz Chan
	Eric Dumonteil
**Additional Information:**	
**Question**	**Response**
**Financial Disclosure**<br><br>Enter a financial disclosure statement that describes the sources of funding for the work included in this submission. Review the submission guidelines for detailed requirements. View published research articles from *PLOS ONE* for specific examples.	This work was supported by Fundación Carlos Slim (#87714) and Consejo Nacional de Ciencia y Tecnología, México (CONACYT, #PDCPN2015-102). B.C.A.A was supported by scholarship #334483 from CONACYT.

Powered by Editorial Manager® and ProduXion Manager® from Aries Systems Corporation

This statement is required for submission and **will appear in the published article** if the submission is accepted. Please make sure it is accurate.<br><br>**Unfunded studies**<br>Enter: *The author(s) received no specific funding for this work.*<br><br>**Funded studies**<br>Enter a statement with the following details:<br>• Initials of the authors who received each award<br>• Grant numbers awarded to each author<br>• The full name of each funder<br>• URL of each funder website<br>• Did the sponsors or funders play any role in the study design, data collection and analysis, decision to publish, or preparation of the manuscript?<br>• **NO** - Include this sentence at the end of your statement: *The funders had no role in study design, data collection and analysis, decision to publish, or preparation of the manuscript.*<br>• **YES** - Specify the role(s) played.<br><br>* typeset	
**Competing Interests**<br><br>Use the instructions below to enter a competing interest statement for this submission. On behalf of all authors, disclose any competing interests that could be perceived to bias this work—acknowledging all financial support and any other relevant financial or non-financial competing interests.<br><br>This statement is required for submission and **will appear in the published article** if the submission is accepted. Please make sure it is accurate and that any funding sources listed in your Funding Information later in the submission form are also declared in your Financial Disclosure statement.<br><br>View published research articles from *PLOS ONE* for specific examples.	The authors have declared that no competing interests exist.

Powered by Editorial Manager® and ProduXion Manager® from Aries Systems Corporation

**NO authors have competing interests**<br><br>Enter: *The authors have declared that no competing interests exist.*<br><br>**Authors with competing interests**<br><br>Enter competing interest details beginning with this statement:<br><br>*I have read the journal's policy and the authors of this manuscript have the following competing interests: [insert competing interests here]*<br><br>* typeset	
**Ethics Statement**<br><br>Enter an ethics statement for this submission. This statement is required if the study involved:<br><br>• Human participants<br>• Human specimens or tissue<br>• Vertebrate animals or cephalopods<br>• Vertebrate embryos or tissues<br>• Field research<br><br>Write "N/A" if the submission does not require an ethics statement.<br><br>General guidance is provided below. Consult the submission guidelines for detailed instructions. **Make sure that all information entered here is included in the Methods section of the manuscript.**	All experimental protocols were approved by the institutional bioethics committee of the "Centro de Investigaciones Regionales Dr. Hideyo Noguchi", Universidad Autónoma de Yucatán (Reference #CEI-08-2019) and were performed in strict compliance with the Official Mexican Standards (NOM-062-ZOO-1999).

Powered by Editorial Manager® and ProduXion Manager® from Aries Systems Corporation

**Format for specific study types**<br><br>**Human Subject Research (involving human participants and/or tissue)**<br>• Give the name of the institutional review board or ethics committee that approved the study<br>• Include the approval number and/or a statement indicating approval of this research<br>• Indicate the form of consent obtained (written/oral) or the reason that consent was not obtained (e.g. the data were analyzed anonymously)<br><br>**Animal Research (involving vertebrate animals, embryos or tissues)**<br>• Provide the name of the Institutional Animal Care and Use Committee (IACUC) or other relevant ethics board that reviewed the study protocol, and indicate whether they approved this research or granted a formal waiver of ethical approval<br>• Include an approval number if one was obtained<br>• If the study involved *non-human primates*, add *additional details* about animal welfare and steps taken to ameliorate suffering<br>• If anesthesia, euthanasia, or any kind of animal sacrifice is part of the study, include briefly which substances and/or methods were applied<br><br>**Field Research**<br><br>Include the following details if this study involves the collection of plant, animal, or other materials from a natural setting:<br>• Field permit number<br>• Name of the institution or relevant body that granted permission	
**Data Availability**<br><br>Authors are required to make all data underlying the findings described fully available, without restriction, and from the time of publication. PLOS allows rare exceptions to address legal and ethical concerns. See the PLOS Data Policy and FAQ for detailed information.	Yes - all data are fully available without restriction

Powered by Editorial Manager® and ProduXion Manager® from Aries Systems Corporation

A Data Availability Statement describing where the data can be found is required at submission. Your answers to this question constitute the Data Availability Statement and **will be published in the article**, if accepted.<br><br>**Important:** Stating 'data available on request from the author' is not sufficient. If your data are only available upon request, select 'No' for the first question and explain your exceptional situation in the text box.<br><br>Do the authors confirm that all data underlying the findings described in their manuscript are fully available without restriction?	
**Describe where the data may be found in full sentences. If you are copying our sample text, replace any instances of XXX with the appropriate details.**<br><br>• If the data are **held or will be held in a public repository**, include URLs, accession numbers or DOIs. If this information will only be available after acceptance, indicate this by ticking the box below. For example: *All XXX files are available from the XXX database (accession number(s) XXX, XXX.).*<br>• If the data are all contained **within the manuscript and/or Supporting Information files**, enter the following: *All relevant data are within the manuscript and its Supporting Information files.*<br>• If neither of these applies but you are able to provide **details of access elsewhere**, with or without limitations, please do so. For example:<br><br>*Data cannot be shared publicly because of [XXX]. Data are available from the XXX Institutional Data Access / Ethics Committee (contact via XXX) for researchers who meet the criteria for access to confidential data.*<br><br>*The data underlying the results presented in the study are available from (include the name of the third party*	All relevant data are within the manuscript and its Supporting Information files.

Powered by Editorial Manager® and ProduXion Manager® from Aries Systems Corporation

and contact information or URL).<br>• This text is appropriate if the data are owned by a third party and authors do not have permission to share the data.<br><br>* typeset	
Additional data availability information:	Tick here if your circumstances are not covered by the questions above and you need the journal's help to make your data available.

Powered by Editorial Manager® and ProduXion Manager® from Aries Systems Corporation

Manuscript
Click here to access/download;Manuscript;July 31 PONE-D-23_22437updated.docx

Full title: Signature of cardiac alterations in early and late chronic infections with *Trypanosoma cruzi* in mice

Short title: Cardiac alterations in *Trypanosoma cruzi* infected-mice

Bárbara Carolina Arias-Argáez[1], Victor Manuel Dzul-Huchim[1], Ana Paulina Haro-Álvarez[2], Miguel Enrique Rosado-Vallado [1], Liliana Villanueva-Lizama [1], Julio Vladimir Cruz-Chan [1*¶], Eric Dumonteil [3*¶]

[1] Laboratorio de Parasitología, Centro de Investigaciones Regionales "Dr. Hideyo Noguchi", Universidad Autónoma de Yucatán, Mérida,Yucatán, México.

[2] Instituto de Investigaciones en Ciencias Veterinarias, Universidad Autónoma de Baja California, Mexicali, Baja California, México.

[3] Department of Tropical Medicine, School of Public Health and Tropical Medicine, and Vector-Borne and Infectious Disease Research Center, Tulane University, New Orleans, Louisiana, USA.

* **Corresponding authors:**

E-mail: vladimir.cruz@correo.uady.mx

edumonte@correo.uady.mx

¶ These authors contributed equally to this work.

Abstract

Chagas disease by *Trypanosoma cruzi* (*T. cruzi*) infection is a leading cause of myocarditis worldwide. Chagas cardiomyopathy is presented with a wide variety of conduction abnormalities including arrhythmias, first- and second-degree atrioventricular blockade, left ventricular systolic dysfunction and some cases heart failure leading to the death. Currently, there are no effective treatments available against advanced Chagas disease. With the advance in the development of novel therapies, it is important to utilize an animal model that can effectively replicate the diverse stages of Chagas disease, including chronic asymptomatic and symptomatic infection, that are akin to those observed in humans. Therefore, to characterize the cardiac alterations during the evolution of the infection, we evaluated the progression of cardiomyopathy caused by *T. cruzi* H1 infection in both Balb/c and ICR mouse models by performing electrocardiogram (ECG) studies in unanesthetized mice every month until 210 days post-infection (dpi). In the late chronic phase of infection, we also performed echocardiogram (ECHO) studies to further assess cardiac function. We demonstrate that ICR mice were more susceptible to cardiac alterations compared to Balb/c mice and suitable as an experimental chronic infection model.

Keywords: Chagas disease, *Trypanosoma cruzi*, Electrocardiography, Echocardiography, Chronic infection.

Introduction

Chagas disease is a worldwide health problem caused by the protozoan parasite *Trypanosoma cruzi* (*T. cruzi*) with 6-7 million people infected worldwide, mainly settled in the Americas [1] The disease initiates with an acute phase which lasts a few weeks and is characterized by parasites in the bloodstream and unspecific symptoms such as nausea, headache, and fever. Next, infected individuals progress into the symptomatic or asymptomatic chronic phase, where the latter is delimited by the absence of abnormalities in the physical examination and cardiac monitoring, while symptomatic individuals, about 30 to 40% of cases, evolve to megaesophagus, megacolon, and cardiomyopathy years after the infection [2, 3].

It is widely acknowledged that the pathogenesis of cardiomyopathy in Chagas disease is predominantly attributed to the persistent presence of *T. cruzi* within cardiac tissue, leading to inflammation and fibrosis. These changes culminate in structural alterations to the cardiac conduction system, resulting in the development of cardiac rhythm abnormalities such as bradyarrhythmia and tachyarrhythmia which in some severe cases, can progress to heart failure [4]. In the early chronic phase infected human patients exhibit abnormalities in myocardial contractility and left ventricular wall motion, which generally are associated with ventricular arrhythmia and cardiac fibrosis areas. [3, 5-7]. Studies in murine models infected with *T. cruzi* have reported differences in cardiac parameters performed by electrocardiogram (ECG) and echocardiogram (ECHO) recordings compared to naive mice. BALB/c mice infected with *T. cruzi* Tulahuen strain developed alterations in the QRS complex as well as prolonged QT intervals in the first 5 weeks of infection, and these

alterations were maintained during the early chronic phase at 90 days post-infection (dpi) [8]. Other alterations have been reported in Balb/c mice infected with *T. cruzi* Colombian strain, where mice displayed prolonged P, PR, and QTc wave intervals, as well decreased heart rate. In addition, an increase in the right ventricle area, a reduction in the ejection fraction and elevated systolic volume in the left ventricle were observed by ECHO assays, during the early chronic phase at 90 dpi [9]. The longest study for chronic infection was performed in ICR mice infected with *T. cruzi* Brazilian strain at 150 dpi which showed a reduction in HR, left ventricular systolic function, and right/left ventricular dilation by ECHOs [10].

The chemotherapy of *T. cruzi* infections is based on nitrofurans and nitroimidazoles which are unsatisfactory since both compounds have toxic-side effects, ineffective in the chronic phase, and are associated with drug resistance [11, 12]. An alternative approach is the development of a vaccine, which could be administered as immunotherapy either to individuals with acute or chronic infection to prevent the development of cardiomyopathy [13-18]. Nevertheless, despite efforts, a vaccine is still in preclinical studies and given the difficulties to perform non-human primates studies, there is an urgent need to optimize an adequate animal model that reproduces ECG and ECHO abnormalities which are the signature of chagasic cardiomyopathy [19]. Thus, we aimed here to characterize the progression of chronic chagasic cardiomyopathy in experimentally infected mice based on ECG and ECHO findings up to 210 dpi.

Material and methods

Ethical standards

All experimental protocols were approved by the institutional bioethics committee of the “Centro de Investigaciones Regionales Dr. Hideyo Noguchi”, Universidad Autónoma de

Yucatán (Reference #CEI-08-2019) and were performed in strict compliance with the Official Mexican Standards (NOM-062-ZOO-1999).

Mice and Parasites

Female Balb/c (n=13) and ICR (n=13) mice were obtained at 4–5 weeks old (Envigo, México) and infected with *T. cruzi* H1 strain, originally isolated from a human case in Yucatán-México and maintained by serial passage in Balb/c mice as previously described [20]. Control groups included non-infected Balb/c (n=3) and ICR (n=3) mice. All animals were housed on a 12-hour light/dark cycle in groups of 3-5 per cage attached with Smart Flow ventilation system (Tecniplast) and air change of 75 times per hour. Animals received food and water *ad libitum* by staff with more than 3 years of experience in animal care. Mice health and behavior were monitored daily, and cages were cleaned weekly. In order to minimize animal stress, we provide them of paper and cardboard tubes previously sterilized by ultra-violet light. Survival was recorded up to 210 dpi which was the end of the study. The euthanasia was performed by deep anesthesia induced with xylazine/ketamine (10mg/kg / 100mg/kg) followed by cervical dislocation. Weight loss, poor body skin or fur condition were considered as sign of endpoint criteria.

Electrocardiography

Electrical cardiac activity was recorded from non-anesthetized infected mice every month until 210 dpi using ECGenie equipment (Mouse Specifics Inc.). Mice were subjected to an adaptation period to minimize stress in the electrocardiograph plate by placing them for 10 min before recordings. The ECGs trace was obtained with standard lead (dipolar lead DII) and amplitude settings to give 2 mV/1 msec. Typically, 20–25 well-defined successive beats were analyzed from each tracing using the EzCG Signal Analysis Software package (Mouse Specifics Inc.). The following ECG-wave parameters were obtained: heart rate (HR), RR interval, PQ interval, PR interval, QT interval, QTc interval, QRS complex, and ST segment.

Echocardiography

To induce anesthesia in mice, isoflurane was administered at a dosage of 3% along with 0.5 L/min O_2 in an anesthetic chamber (Patterson Scientific). The mice were then maintained at a dosage of 1.5-2.5% isoflurane using a face mask. Echocardiography (ECHO) recordings were obtained using a 22 MHz linear-array ultrasound transducer of the Mylab Seven system (Esaote Inc.). Mice were placed in a supine position on a heated surgical platform (Indus Instruments) maintained at a temperature of 37°C and excessive hair was removed with depilatory cream (NairTM). The short-axis images were obtained of the left ventricle (LV) in B and M modes at the papillary muscle level. The systolic function of LV was evaluated by measuring left ventricular ejection fraction (LVEF). The formula to calculate LVEF was: LVEF(%)= [(LVEDV-LVESV)/LVEDV] x 100, where LVEDV and LVESV= LV end-diastolic/systolic volume [21].

Data and statistical analysis

The parasitemia and ECG parameters from each time point were analyzed by student's t-test and survival curves with the Mantel-Cox Long-rank test. A P-value less than 0.05 was considered significant statistically. The ECG parameters were also integrated into a multivariate analysis, and we performed Linear Discriminant Analysis (LDA) to evaluate the effect of *T. cruzi* infection on ECG patterns. The statistical significance of differences was assessed by Permutational Multivariate Analysis of Variance (PERMANOVA) and Bonferroni correction to adjust *P* values for multiple pairwise comparations. We further combined ECG and ECHO parameters into an LDA analysis of cardiac function.

Results

Susceptibility of mice to *T. cruzi* infection

To follow the progression of the *T. cruzi* infection towards the chronic phase, the blood parasite burden and survival were measured in both Balb/c and ICR mice infected with 500

blood trypomastigotes from a considered lethal dose of infection with the *T. cruzi* H1 strain. At 23 dpi, we observed that the levels of parasitemia were significantly higher in ICR compared to Balb/c mice (**Fig 1A**). This data was correlated with survival, as ICR mice showed high mortality 5/13 (38.47%) starting at 27 dpi and ending at day 32, while in Balb/c strain there was no mortality. Thus, all Balb/c mice survived up to 210 dpi and only 8/13 (61.53%) of ICR mice survived infection (**Fig 1B**). As expected, no mortalities were observed in non-infected control groups during that time. None mice showed sign of endpoint criteria.

Figure 1. Parasitemia and survival in *T. cruzi*-infected mice. Balb/c (n=13) and ICR (n=13) mice were infected with 500 blood trypomastigotes of H1 *T. cruzi* by intraperitoneal injection. A total of 3 mice were used as the non-infected control group for each mouse strain and received only saline solution. **A)** Parasitemia is shown as mean ± S.D. Significant differences were calculated with the Mann-Whitney´s U-test, and is indicated as follow *, $P \leq 0.05$. **B)** Survival was monitored during 210 dpi. Significant differences were calculated using Mantel-Cox log-rank test, *, $P<0.05$.

Progression of ECG alterations in *T. cruzi* infected mice

Electrical cardiac activity in non-anesthetized ICR and Balb/c mice showed differences in the infection by *T. cruzi* (from 0 to 210 dpi.). Among the significant cardiac alterations found in infected ICR mice, we observed that 100% (8/8) of mice at 37 dpi showed a decreased HR, which was correlated with an increase in the ST segment (**Fig 2A, G**). In addition, at the beginning of the chronic phase at 70 dpi, 87.5% (7/8) of infected ICR mice showed a decreased HR and by 105 dpi PQ and PR intervals as well as ST segment were increased compared to non-infected ICR mice (**Fig 2C, E, G**). In the late chronic phase, 87.5% (7/8) of infected ICR mice also showed an increased PQ and PR intervals at 175 dpi (**Fig 2C, E**) and an increased ST segment at 210 dpi compared to non-infected mice (**Fig 2G**). All this data suggests that *T. cruzi* H1 strain changes the cardiac electrical activity in ICR mice throughout infection. In contrast, no significant difference in any of the ECG parameters

measured was detected in infected Balb/c mice compared to those uninfected during *T. cruzi* infection (**Fig 2B, D, F, H).**

Figure 2. ECGs from non-infected (white dots) and infected mice (marron and blue dots for ICR and Balb/c, respectively). ECGs were recorded monthly from day 0 (before infection) to 210 dpi. The following recordings were **A, B)** Heart rate, **C, D)** PQ interval, **E, F)** PR interval and **G, H)** ST interval. Data are shown as mean ± SEM. Significant differences were estimated with Student´s t-test comparing infected and non-infected mice, and is indicated as follows *, $P \leq 0.05$.

Multivariate analysis of ECG parameters during *T. cruzi* infection

We then integrated ECG parameters into a multivariate analysis to assess global changes in ECG patterns in response to *T. cruzi* infection during the acute phase of infection (0, 37 and 70 dpi) compared to the early (105 and 140 dpi) and late chronic phases (175 and 210 dpi) through Linear Discriminant Analysis (LDA) for both ICR and Balb/c mice. A significant effect of *T. cruzi* infection on ECG patterns was detected in both mouse strains (PERMANOVA F=2.4, P=0.033 for Balb/c and F=8.23, P=0.0003 for ICR mice, respectively). However, the time course of cardiac alterations differed between Balb/c and ICR mice. Indeed, combined ECG alterations observed in Balb/c mice during the acute phase of *T. cruzi* infection were contrasting compared to the chronic late phase (**Fig 3A and Table 1**). Conversely, ICR mice showed significant ECG alterations in all stages of infection (acute, early, and late chronic) with increased deviation from normal ECG patterns from uninfected mice over time (**Fig 3B and Table 1**). Accordingly, the reclassification of individual mice into their respective groups based on their ECG patterns was correct for only 45.9% of Balb/c mice, although it reached 61.5% for mice in the acute phase of *T. cruzi* infection. Reclassification was more accurate for ICR mice, reaching an average of 60.5% correct reclassification, and uninfected and late chronically infected mice were best identified with

76.4% and 75.0% correct reclassification, respectively (**S1 Table**). We then incorporated ECHO data from 210 dpi for the analysis of cardiac alterations in chronically infected mice. As shown in **Fig 4**, there was a major effect caused by *T. cruzi* infection on cardiac function from ICR compared to Balb/c mice in the late chronic phase. Overall, 87.5% of mice could be correctly reclassified according to their combined ECHO and ECG parameters (**S2 Table**).

Figure 3. Effect of *T. cruzi* infection on the ECG profiles of Balb/c and ICR mice. ECG parameters were analyzed by LDA/PERMANOVA. **A)** Balb/c mice presented significant differences in ECG patterns following *T. cruzi* infection (PERMANOVA, *P*=0.03), mostly during the acute phase of infection (*P*=0.021). **B)** ICR mice also presented significant differences in ECG patterns following *T. cruzi* infection (PERMANOVA, *P*=0.0003), for all periods (*P*=0.031, *P*=0.003 and *P*=0.0006 for acute, early, and late chronic phases vs uninfected saline control, respectively. Chr: chronic.

Figure 4. Effect of *T. cruzi* infection on ECG and ECHO profiles of Balb/c and ICR mice during the late chronic phase. Data were analyzed by LDA/PERMANOVA. Significant ECG and ECHO alterations for ICR mice at 210 days post-infection (PERMANOVA, *P*=0.009) indicated more severe cardiac disease in ICR mice compared to Balb/c mice.

Table 1. Pairwise comparisons of ECG patterns in *T. cruzi* infected mice. Bonferroni-adjusted *P* values for pairwise comparisons are indicated for Balb/c mice (Above the diagonal) and for ICR mice (Below the diagonal). * Indicates a statistically significant difference.

	Uninfected	Acute	Early Chronic	Late Chronic
**Uninfected**	-	0.95	1	0.92
**Acute**	0.031*	-	0.27	0.021*
**Early Chronic**	0.003*	1	-	1
**Late Chronic**	0.0006*	0.97	1	-

Discussion

As Chagas disease remains a public health problem, so has the milestone of developing a vaccine to halt or delay the pathology by *T. cruzi* infection [14, 16-18, 22]. Thus, an adequate murine model plays a key for the discovery and testing of new antigens in experimental studies. Previously, Balb/c and ICR mice have been used for the study of both acute and chronic phases of experimental *T. cruzi* infection [23-26]; however, there are scarce studies focused on cardiac alterations in chronically *T. cruzi*-infected mice. Hence, in this study, we report for the first time a multi-variate analysis of the comprehensive cardiac pathology parameters in *T. cruzi*-infected Balb/c and ICR mice, indicating major differences in the progression and severity of cardiac pathology depending on the mouse strain. Our results could contribute to the selection of an adequate murine model for the study of new immunotherapies against chronic Chagas disease using a lethal/sub-lethal infective-dose for Balb/c and ICR mice by inoculation of 500 blood trypomastigotes of *T. cruzi* H1 strain, according to previous studies [20]. Our data indicate that ICR mouse strain was more susceptible to *T. cruzi* H1 infection, showing a higher blood parasite burden and decreased survival compared to Balb/c mice infected with the same parasite dose.

In this study, we assessed the cardiac electrophysiology of unanesthetized mice during experimental *T. cruzi* H1 infection using electrocardiograms (ECGs). It was essential to evaluate the cardiac function of unanesthetized animals, as the administration of anesthetics has to decrease the LV systolic function, HR, as well as blood pressure depending on the type of anesthetic used [10, 27, 28]. ECGs recordings showed that during *T. cruzi* infection, Balb/c mice did not exhibit electrical alterations, conversely to ICR mice which showed altered electrocardiographic parameters for all stages of infection. Upon the early stages of chronic *T. cruzi* infection, ICR mice exhibited a reduction in HR, suggestive of sinus bradycardia, at 37 and 70 dpi. However, from 105 dpi onward, infected mice demonstrated normalization of HR, indicating a potential compensatory mechanism to

preserve cardiac function. At the early (105 dpi) and late (175 dpi) chronic phase, prolonged PQ and PR intervals associated with first-degree AV blockade were observed in infected ICR mice but not in Balb/c mice. In humans, chagasic patients with positive serology from rural communities in Brazil and Bolivia also showed longer PQ and PR intervals, and as a consequence of these conduction disturbances patients develop first- and second-degree atrioventricular blocks [29]. The first and second-degree atrioventricular blocks are abnormalities found in *T. cruzi*-infected dogs during the acute or chronic phase [30, 31]. Likewise, first-degree atrioventricular conduction disturbance, systolic dysfunction, and ventricular arrhythmias are also ECG abnormalities found in rhesus macaques (Macaca mulatta) chronically infected with *T. cruzi* Colombian strain. The electrical disturbances observed in Chagasic patients as right bundle branch block, ventricular tachycardia, second or third-degree atrioventricular block, left bundle branch block, and atrial fibrillation [32-34] are consistent with our findings in ICR mice that appear to be developing chronic chagasic cardiomyopathy. Myocardial ischemic is also a common anomaly detected in chronic Chagasic patients with heart disease, as well as in naturally infected dogs during early and late stages of infection, with most presenting a longer ST segment [35-37]. Here, infected ICR mice also showed prolonged ST segment, suggesting myocardial ischemia in acute, early, and late chronic infection, but additionally, sinus bradycardia in the acute infection and atrioventricular blockade in early and late chronic phases whereas ECGs of Balb/c mice revealed no changes in conduction abnormalities during all stages of *T. cruzi* infection.

ECHOs further revealed a more severe dysfunction in the ejection fraction from ICR mice compared to Balb/c in the late chronic phase at 210 dpi. Consistent with our data, prior investigations of cardiac function in Balb/c mice infected with *T. cruzi* H1 strain revealed a steady ejection fraction at 208 dpi of 54.76% ± 9.35 compared to naïve mice with 48.33% ± 9 [38]. Similarly, *T. cruzi*-infected Balb/c mice exhibited an ejection fraction during chronic infection at 212 dpi with no significant differences with control mice [39].

A limitation of this study is that we could not measure fibrosis and inflammatory cell infiltration in the cardiac tissue of the infected mice. However, a previous study from our group showed cardiac fibrosis and inflammation after 200 dpi in Balb/c mice infected with the *T. cruzi* H1 strain [38, 39]. Hence, we speculate that cardiac alterations evaluated by ECGs and ECHOs may be a result of cardiac fibrosis and inflammation caused by *T. cruzi* H1 infection. Additionally, a previous study using Brazil, Tulahuen, or Sylvio-X10/4 *T. cruzi* strains suggested that ECGs can be used as a non-invasive method to screen chronic histopathology damage in infected Balb/c mice [8].

In the last years, LDA/PERMANOVA analysis has been used as a robust method to integrate multiple variables to evaluate associations in many biological, ecological, and environmental data sets [40, 41]. In ECG data analysis, the use of univariate statistics was unable to identify significant differences in any of these parameters, hence, multivariate analysis had a greater power to identify statistical changes [42]. Our analysis allowed us to estimate the effect of *T. cruzi* infection on all ECGs-data recordings together in both murine models. Our findings align well with the three phases of *T. cruzi* infection in both murine models: an acute phase (0-70 dpi), an early chronic phase (105-140 dpi), and a late chronic phase (175-210 dpi) and highlight clear differences in the progression of cardiac disease in these two mouse strains.

In sum, we demonstrated that the ICR mouse strain is significatively more susceptible to *T. cruzi* H1 infection than Balb/c mice, and it develops more severe cardiac disease. This study supports the use of both mouse strains as suitable experimental models to study chronic *T. cruzi* infection and novel treatments, as each strain presents different profiles of cardiac alterations that are consistent with the well-characterized variability observed in human Chagasic cardiomyopathy.

Author contributions

Conceptualization: Bárbara Carolina Arias-Argáez, Ana Paulina Haro-Álvarez, Miguel Enrique Rosado-Vallado, Julio Vladimir Cruz-Chan, Eric Dumonteil.

Data curation: Bárbara Carolina Arias-Argáez, Julio Vladimir Cruz-Chan, Eric Dumonteil.

Formal analysis: Bárbara Carolina Arias-Argáez, Julio Vladimir Cruz-Chan, Eric Dumonteil.

Funding acquisition: Ana Paulina Haro-Álvarez, Miguel Enrique Rosado-Vallado, Julio Vladimir Cruz-Chan, Eric Dumonteil.

Investigation: Victor Manuel Dzul-Huchim, Liliana Villanueva-Lizama.

Methodology: Bárbara Carolina Arias-Argáez, Ana Paulina Haro-Álvarez, Miguel Enrique Rosado-Vallado, Julio Vladimir Cruz-Chan, Eric Dumonteil.

Resources: Victor Manuel Dzul-Huchim, Liliana Villanueva-Lizama.

Supervision: Ana Paulina Haro-Álvarez, Miguel Enrique Rosado-Vallado, Julio Vladimir Cruz-Chan, Eric Dumonteil.

Visualization: Bárbara Carolina Arias-Argáez, Ana Paulina Haro-Álvarez, Miguel Enrique Rosado-Vallado, Julio Vladimir Cruz-Chan, Eric Dumonteil.

Writing – original draft: Bárbara Carolina Arias-Argáez, Victor Manuel Dzul-Huchim

Writing – review & editing: Julio Vladimir Cruz-Chan, Eric Dumonteil.

Financial Support

This work was supported by Fundación Carlos Slim (#87714) and Consejo Nacional de Ciencia y Tecnología, México (CONACYT, #PDCPN2015-102). B.C.A.A was supported by scholarship #334483 from CONACYT.

References

1. GBD 2015 Disease and Injury Incidence and Prevalence Collaborators. Global, regional, and national incidence, prevalence, and years lived with disability for 310 diseases and injuries, 1990-2015: a systematic analysis for the Global Burden of Disease Study 2015. Lancet. 2016;388(10053): 1545-602. doi:10.1016/S0140-6736(16)31678-6. PMID: 27733282.

2. Teixeira MM, Gazzinelli RT, Silva JS. Chemokines, inflammation and *Trypanosoma cruzi* infection. Trends Parasitol. 2002;18(6): 262-5. doi:10.1016/s1471-4922(02)02283-3. PMID: 12036740.

3. Ribeiro AL, Nunes MP, Teixeira MM, Rocha MO. Diagnosis and management of Chagas disease and cardiomyopathy. Nat Rev Cardiol. 2012;9(10): 576-89. doi:10.1038/nrcardio.2012.109. PMID: 22847166.

4. Rassi A, Jr., Marin JAN, Rassi A. Chronic Chagas cardiomyopathy: a review of the main pathogenic mechanisms and the efficacy of aetiological treatment following the BENznidazole Evaluation for Interrupting Trypanosomiasis (BENEFIT) trial. Mem Inst Oswaldo Cruz. 2017;112(3): 224-35. doi:10.1590/0074-02760160334. PMID: 28225900.

5. Viotti RJ, Vigliano C, Laucella S, Lococo B, Petti M, Bertocchi G, et al. Value of echocardiography for diagnosis and prognosis of chronic Chagas disease cardiomyopathy without heart failure. Heart. 2004;90(6): 655-60. doi:10.1136/hrt.2003.018960. PMID: 15145872.

6. Barros ML, Ribeiro A, Nunes Mdo C, Rocha MO. Association between left ventricular wall motion abnormalities and ventricular arrhythmia in the indeterminate form of Chagas disease. Rev Soc Bras Med Trop. 2011;44(2): 213-6. doi:10.1590/s0037-86822011005000020. PMID: 21503552.

7. Nunes MCP, Beaton A, Acquatella H, Bern C, Bolger AF, Echeverría LE, et al. Chagas Cardiomyopathy: An Update of Current Clinical Knowledge and Management: A

Scientific Statement From the American Heart Association. Circulation. 2018;138(12): e169-e209. doi:10.1161/cir.0000000000000599. PMID: 30354432.

8. Eickhoff CS, Lawrence CT, Sagartz JE, Bryant LA, Labovitz AJ, Gala SS, et al. ECG detection of murine chagasic cardiomyopathy. J Parasitol. 2010;96(4): 758-64. doi:10.1645/ge-2396.1. PMID: 20738200.

9. Vilar-Pereira G, Carneiro VC, Mata-Santos H, Vicentino AR, Ramos IP, Giarola NL, et al. Resveratrol Reverses Functional Chagas Heart Disease in Mice. PLoS Pathog. 2016;12(10): e1005947. doi:10.1371/journal.ppat.1005947. PMID: 27788262.

10. Chandra M, Shirani J, Shtutin V, Weiss LM, Factor SM, Petkova SB, et al. Cardioprotective effects [illegible]erapamil on myocardial structure and function in a murine model of chronic Trypanosoma cruzi infection (Brazil Strain): an echocardiographic study. Int J Parasitol. 2002;32(2): 207-15. doi:10.1016/s0020-7519(01)00320-4. PMID: 11812498.

11. Rodriques Coura J, de Castro SL. A critical review on Chagas disease chemotherapy. Mem Inst Oswaldo Cruz. 2002;97(1): 3-24. doi:10.1590/s0074-02762002000100001. PMID: 11992141.

12. Bustamante JM, Craft JM, Crowe BD, Ketchie SA, Tarleton RL. New, combined, and reduced dosing treatment protocols cure *Trypanosoma cruzi* infection in mice. J Infect Dis. 2014;209(1): 150-62. doi:10.1093/infdis/jit420. PMID: 23945371.

13. Dumonteil E, Bottazzi ME, Zhan B, Heffernan MJ, Jones K, Valenzuela JG, et al. Accelerating the development of a therapeutic vaccine for human Chagas disease: rationale and prospects. Expert Rev Vaccines. 2012;11(9): 1043-55. doi:10.1586/erv.12.85. PMID: 23151163.

14. Pereira IR, Vilar-Pereira G, Marques V, da Silva AA, Caetano B, Moreira OC, et al. A human type 5 adenovirus-based *Trypanosoma cruzi* therapeutic vaccine re-programs immune response and reverses chronic cardiomyopathy. PLoS Pathog. 2015;11(1): e1004594. doi:10.1371/journal.ppat.1004594. PMID: 25617628.

15. Prochetto E, Bontempi I, Rodeles L, Cabrera G, Vicco M, Cacik P, et al. Assessment of a combined treatment with a therapeutic vaccine and benznidazole for the *Trypanosoma cruzi* chronic infection. Acta tropica. 2022;229: 106334. doi:10.1016/j.actatropica.2022.106334. PMID: 35101415.

16. Dzul-Huchim VM, Ramirez-Sierra MJ, Martinez-Vega PP, Rosado-Vallado ME, Arana-Argaez VE, Ortega-Lopez J, et al. Vaccine-linked chemotherapy with a low dose of benznidazole plus a bivalent recombinant protein vaccine prevents the development of cardiac fibrosis caused by *Trypanosoma cruzi* in chronically-infected BALB/c mice. PLoS neglected tropical diseases. 2022;16(9): e0010258. doi:10.1371/journal.pntd.0010258. PMID: 36095001.

17. González-López C, Chen WH, Alfaro-Chacón A, Villanueva-Lizama LE, Rosado-Vallado M, Ramirez-Sierra MJ, et al. A novel multi-epitope recombinant protein elicits an antigen-specific CD8+ T cells response in *Trypanosoma cruzi*-infected mice. Vaccine. 2022;40(45): 6445-9. doi:10.1016/j.vaccine.2022.09.068. PMID: 36184402.

18. Bunkofske ME, Perumal N, White B, Strauch EM, Tarleton R. Epitopes in the Glycosylphosphatidylinositol Attachment Signal Peptide of *Trypanosoma cruzi* Mucin Proteins Generate Robust But Delayed and Nonprotective CD8+ T Cell Responses. J Immunol. 2023. doi:10.4049/jimmunol.2200723. PMID: 36603035.

19. Chatelain E, Scandale I. Animal models of Chagas disease and their translational value to drug development. Expert Opin Drug Discov. 2020;15(12): 1381-402. doi:10.1080/17460441.2020.1806233. PMID: 32812830.

20. Dumonteil E, Escobedo-Ortegon J, Reyes-Rodriguez N, Arjona-Torres A, Ramirez-Sierra MJ. Immunotherapy of *Trypanosoma cruzi* infection with DNA vaccines in mice. Infect Immun. 2004;72(1): 46-53. doi:10.1128/iai.72.1.46-53.2004. PMID: 14688079.

21. Stypmann J, Engelen MA, Troatz C, Rothenburger M, Eckardt L, Tiemann K. Echocardiographic assessment of global left ventricular function in mice. Lab Anim. 2009;43(2): 127-37. doi:10.1258/la.2007.06001e. PMID: 19237453.

22. Teh-Poot C, Tzec-Arjona E, Martínez-Vega P, Ramirez-Sierra MJ, Rosado-Vallado M, Dumonteil E. From genome screening to creation of vaccine against *Trypanosoma cruzi* by use of immunoinformatics. J Infect Dis. 2015;211(2): 258-66. doi:10.1093/infdis/jiu418. PMID: 25070943.

23. Padilla AM, Rosenberg C, Cook P, Sanchez-Valdez F, McElhannon C, Tarleton RL. Delayed Activation of T Cells at the Site of Infection Facilitates the Establishment of *Trypanosoma cruzi* in Both Naive and Immune Hosts. mSphere. 2023;e0060122. doi:10.1128/msphere.00601-22. PMID: 36695605.

24. Rios LE, Lokugamage N, Garg NJ. Effects of Acute and Chronic *Trypanosoma cruzi* Infection on Pregnancy Outcomes in Mice: Parasite Transmission, Mortality, Delayed Growth, and Organ Damage in Pups. Am J Pathol. 2022;193(3): 313-331. doi: 10.1016/j.ajpath.2022.11.010. PMID: 36565805.

25. Barry MA, Versteeg L, Wang Q, Pollet J, Zhan B, Gusovsky F, et al. A therapeutic vaccine prototype induces protective immunity and reduces cardiac fibrosis in a mouse model of chronic *Trypanosoma cruzi* infection. PLoS neglected tropical diseases. 2019;13(5): e0007413. doi:10.1371/journal.pntd.0007413. PMID: 31145733.

26. Cruz-Chan JV, Villanueva-Lizama LE, Versteeg L, Damania A, Villar MJ, González-López C, et al. Vaccine-linked chemotherapy induces IL-17 production and reduces cardiac pathology during acute *Trypanosoma cruzi* infection. Sci Rep. 2021;11(1): 3222. doi:10.1038/s41598-021-82930-w. PMID: 33547365.

27. Berry CJ, Thedens DR, Light-McGroary K, Miller JD, Kutschke W, Zimmerman KA, et al. Effects of deep sedation or general anesthesia on cardiac function in mice undergoing

cardiovascular magnetic resonance. J Cardiovasc Magn Reson. 2009;11(1): 16. doi:10.1186/1532-429x-11-16. PMID: 19454023.

28. Li C, Dai J, Wu F, Zhang H. Impacts of different anesthetic agents on left ventricular systolic function in mice assessed by echocardiography. Physiol Res. 2019;68(3): 365-74. doi:10.33549/physiolres.933940. PMID: 30904003.

29. Williams-Blangero S, Magalhaes T, Rainwater E, Blangero J, Corrêa-Oliveira R, Vandeberg JL. Electrocardiographic characteristics in a population with high rates of seropositivity for *Trypanosoma cruzi* infection. The American journal of tropical medicine and hygiene. 2007;77(3): 495-9. PMID: 17827366.

30. Meyers AC, Ellis MM, Purnell JC, Auckland LD, Meinders M, Saunders AB, et al. Selected cardiac abnormalities in *Trypanosoma cruzi* serologically positive, discordant, and negative working dogs along the Texas-Mexico border. BMC Vet Res. 2020;16(1): 101. doi:10.1186/s12917-020-02322-6. PMID: 32228593.

31. Cruz-Chan JV, Bolio-González M, Colín-Flores R, Ramirez-Sierra MJ, Quijano-Hernandez I, Dumonteil E. Immunopathology of natural infection with *Trypanosoma cruzi* in dogs. Vet Parasitol. 2009;162(1-2): 151-5. doi:10.1016/j.vetpar.2009.02.024. PMID: 19324497.

32. Andrade JP, Marin Neto JA, Paola AA, Vilas-Boas F, Oliveira GM, Bacal F, et al. I Latin American Guidelines for the diagnosis and treatment of Chagas' heart disease: executive summary. Arq Bras Cardiol. 2011;96(6): 434-42. doi: 10.1590/s0066-782x2011000600002. PMID: 21789345.

33. Shikanai-Yasuda MA, Carvalho NB. Oral transmission of Chagas disease. Clin Infect Dis. 2012;54(6): 845-52. doi:10.1093/cid/cir956. PMID: 22238161.

34. Rassi A, Jr., Rassi A, Marin-Neto JA. Chagas disease. Lancet. 2010;375(9723): 1388-402. doi:10.1016/s0140-6736(10)60061-x. PMID: 20399979.

35. Marin-Neto JA, Cunha-Neto E, Maciel BC, Simões MV. Pathogenesis of chronic Chagas heart disease. Circulation. 2007;115(9): 1109-23. doi:10.1161/circulationaha.106.624296. PMID: 17339569.

36. Simões MV, Ayres EM, Santos JL, Schmidt A, Pintya AO, Maciel BC, et al. Detection of myocardial ischemia in chronic Chagas disease patients with atypic precordial pain by exercise and Holter tests. Arq Bras Cardiol. 1993;60(5): 315-9. PMID: 8311747.

37. Montenegro VM, Jimenez M, Dias JC, Zeledon R. Chagas disease in dogs from endemic areas of Costa Rica. Mem Inst Oswaldo Cruz. 2002;97(4): 491-4. doi:10.1590/s0074-02762002000400006. PMID: 12118277.

38. Jones KM, Mangin EN, Reynolds CL, Villanueva LE, Cruz JV, Versteeg L, et al. Vaccine-linked chemotherapy improves cardiac structure and function in a mouse model of chronic Chagas disease. Front Cell Infect Microbiol. 2023;13: 1106315. doi:10.3389/fcimb.2023.1106315. PMID: 36844399.

39. Hoffman KA, Reynolds C, Bottazzi ME, Hotez P, Jones K. Improved Biomarker and Imaging Analysis for Characterizing Progressive Cardiac Fibrosis in a Mouse Model of Chronic Chagasic Cardiomyopathy. J Am Heart Assoc. 2019;8(22): e013365. doi:10.1161/jaha.119.013365. PMID: 31718442.

40. Bourhane Z, Cagnon C, Castañeda C, Rodríguez-Ochoa R, Álvaro-Fuentes J, Cravo-Laureau C, et al. Vertical organization of microbial communities in Salineta hypersaline wetland, Spain. Front Microbiol. 2023;14: 869907. doi:10.3389/fmicb.2023.869907. PMID: 36778872.

41. Dumonteil E, Pronovost H, Bierman EF, Sanford A, Majeau A, Moore R, et al. Interactions among Triatoma sanguisuga blood feeding sources, gut microbiota and *Trypanosoma cruzi* diversity in southern Louisiana. Mol Ecol. 2020;29(19): 3747-61. PMID: 32749727.

42. Dumonteil E, Herrera C, Marx PA. Safety and preservation of cardiac function following therapeutic vaccination against *Trypanosoma cruzi* in rhesus macaques. J Microbiol Immunol Infect. 2023;56(2): 400-7. doi:10.1016/j.jmii.2022.09.003. PMID: 36210315.

Supporting information

S1 Table. Reclassification matrix of individual mice according to LDA analysis of ECG patterns.

S2 Table. Reclassification matrix of individual mice according to LDA analysis of ECG patterns.

 Click here to access/download;Figure;Fig 1.tif

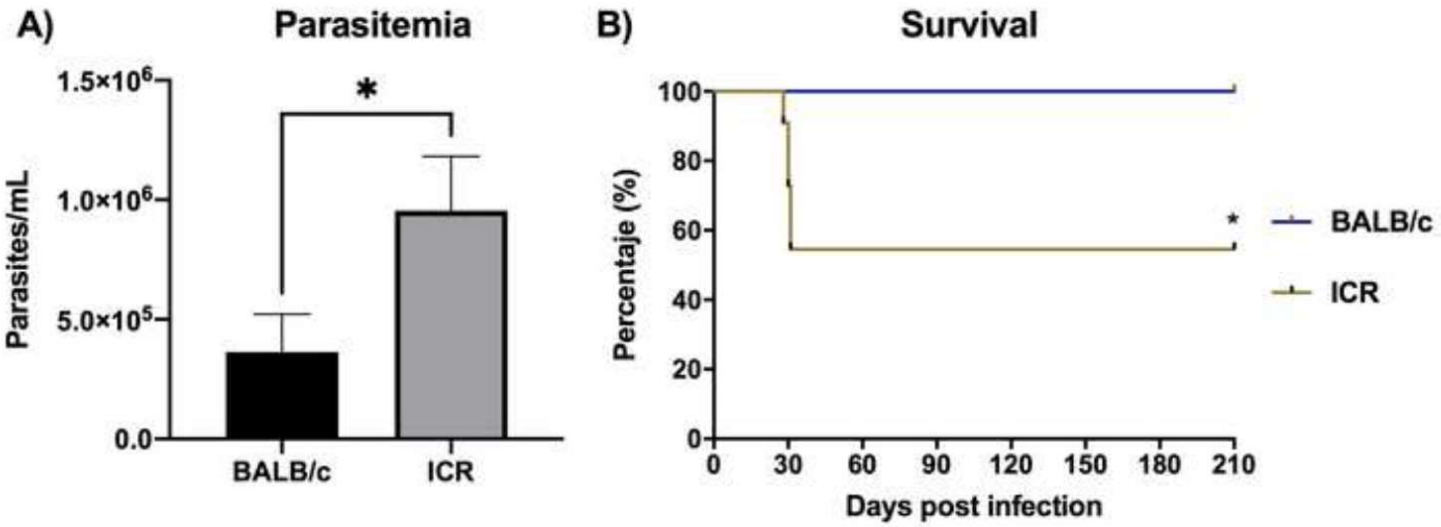

Figure 2

Click here to access/download;Figure;Fig 2.tif

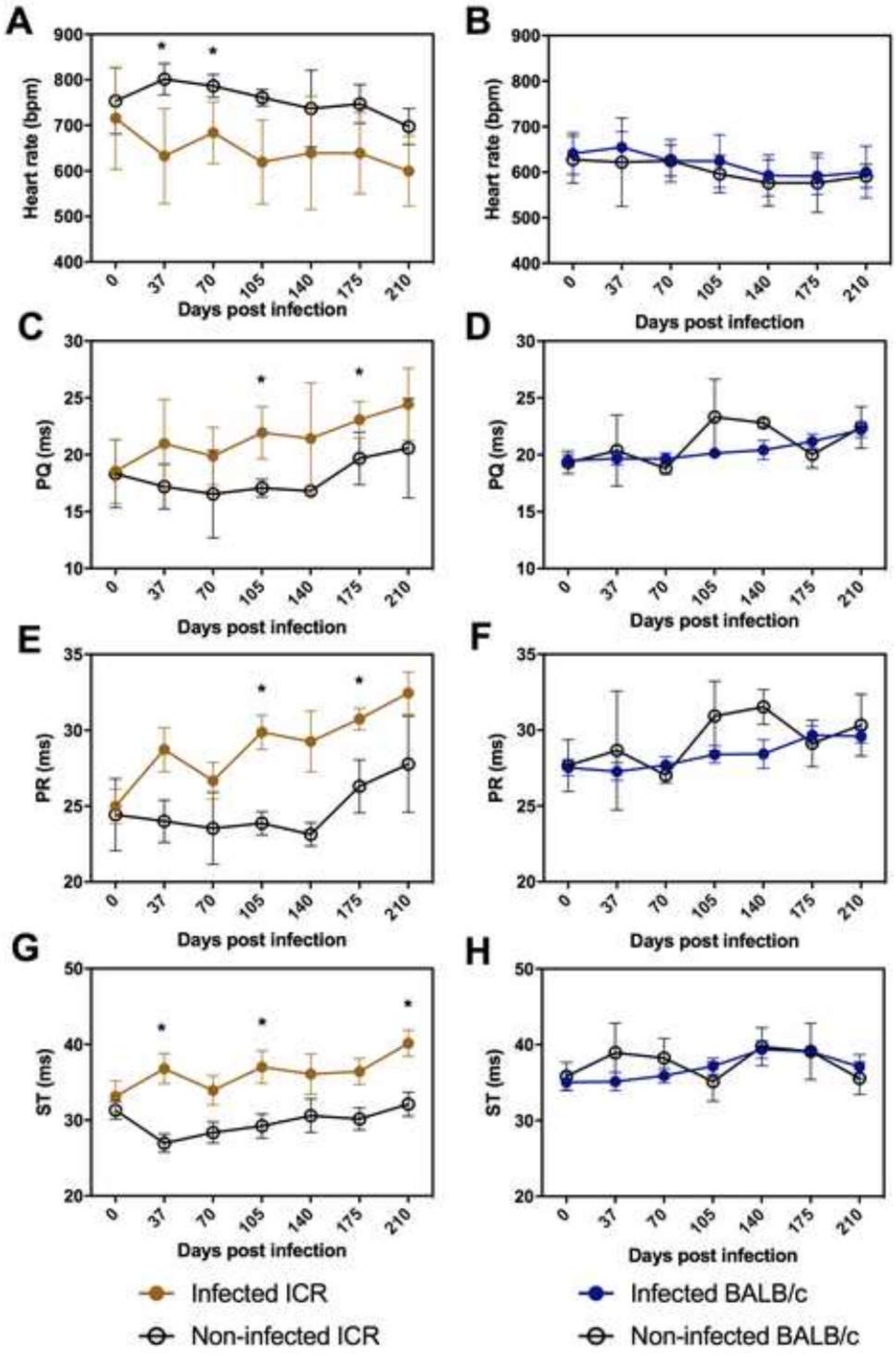

Click here to access/download;Figure;Fig 3.tif

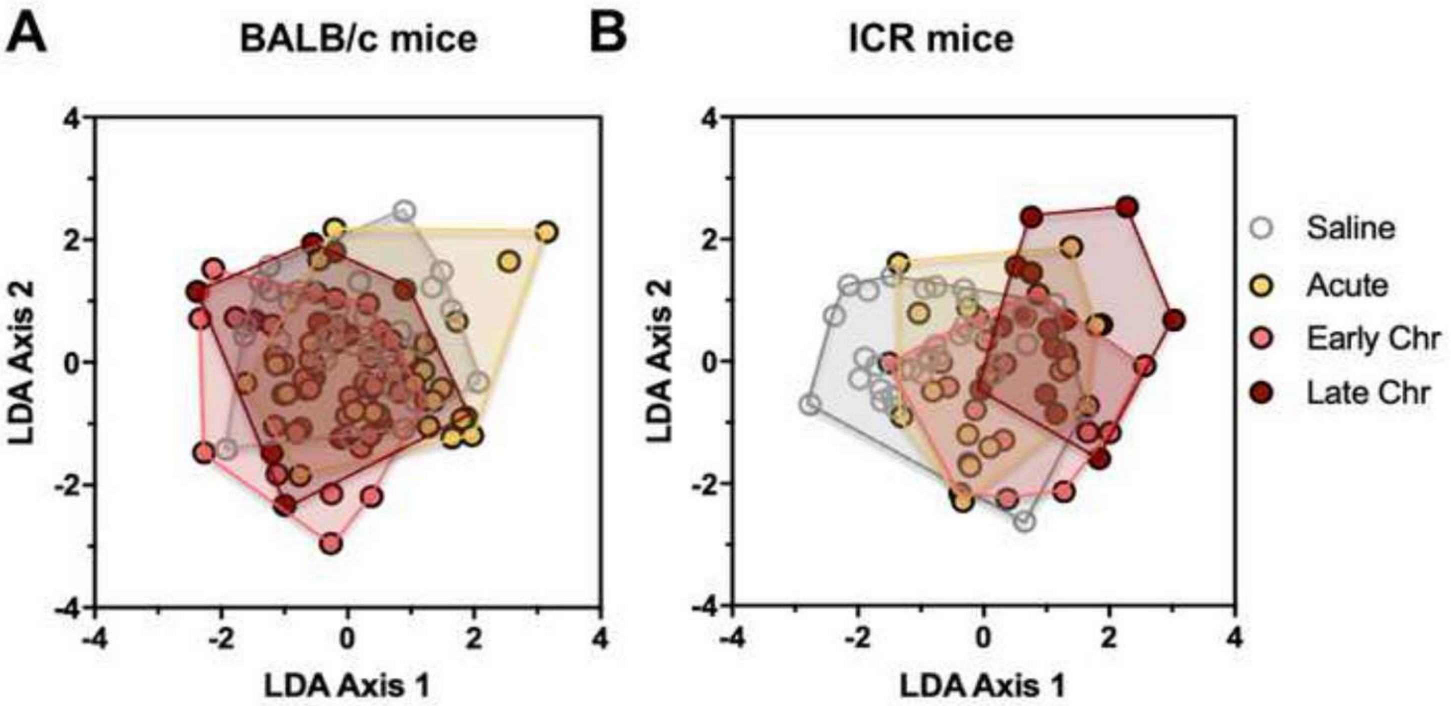

Click here to access/download;Figure;Fig 4.tif

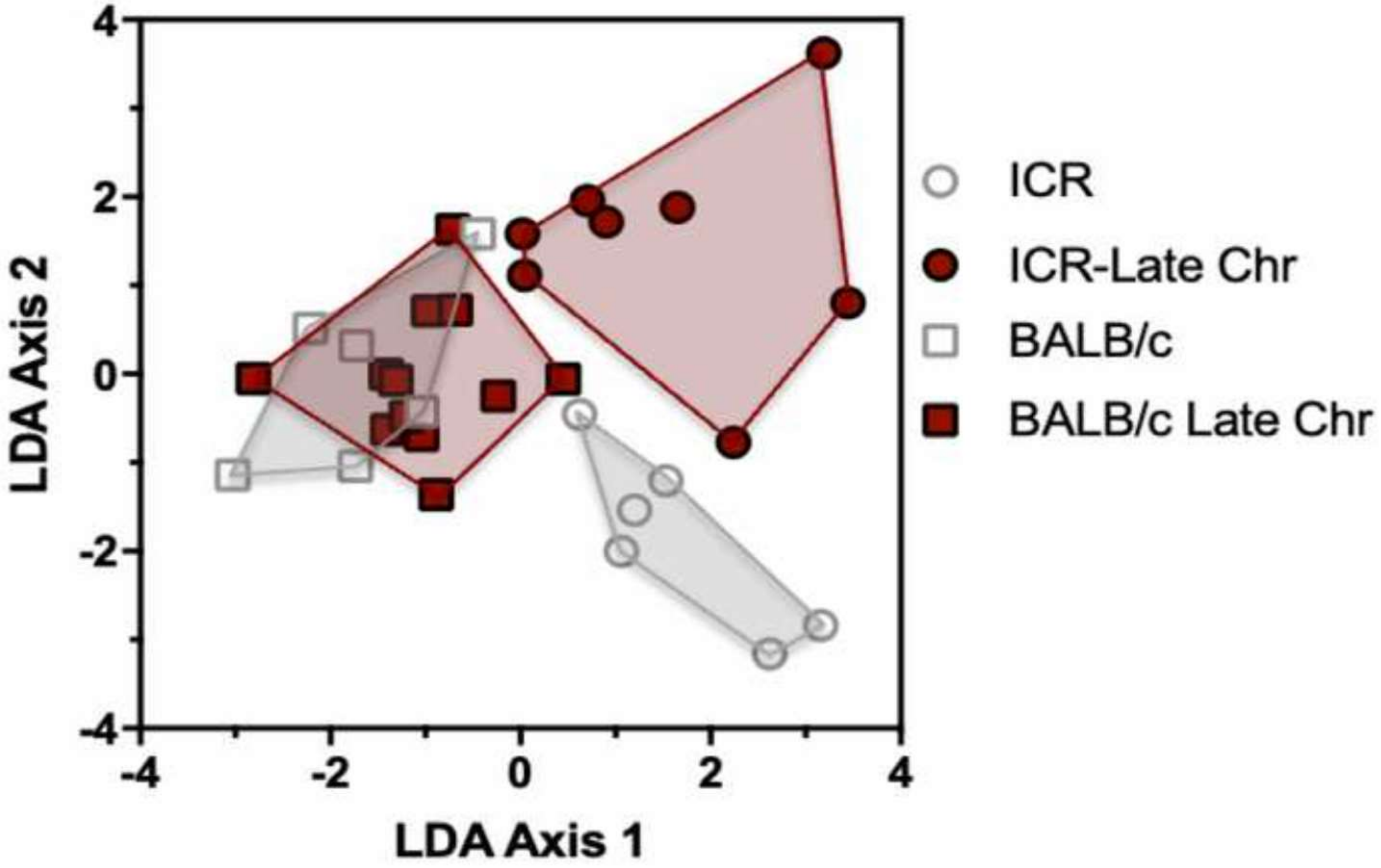

Click here to access/download
Supporting Information
S1 Table.docx

Click here to access/download
Supporting Information
S2 Table.docx
