# Supplementary material for: Signature of cardiac alterations in early and late chronic infections with Trypanosoma cruzi in mice
Source: PLoS One. 2023 Oct 5;18(10):e0292520. doi: 10.1371/journal.pone.0292520 (PMC10553825; doi:10.1371/journal.pone.0292520)
Supplement: Attachment — Submitted filename: Response Letter VC20sep23.docx [file pone.0292520.s004.docx]

**Response to reviewers**

Reviewer #1: The manuscript entitled "Signature of cardiac alterations in early and late chronic infections with Trypanosoma cruzi in mice" evaluated cardiac alterations associated with Chagas disease. I have minor concerns:

1- Add the conclusion to the abstract.

**RESPONSE: *We thank the reviewer 1 for improving the MS.* *We have added the conclusion in the Abstract section. Please see lines 46-50 in Tracked version.***

2- Abbreviations should be defined in their first use. For example, "dpi" should be defined in the main text.

**RESPONSE: *We have revised all the MS correctly writing the abbreviations.***

3- There are several typos and grammar errors in the manuscript. Revise the whole paper in this regard.

**RESPONSE: *We have revised all the MS to avoid typos and grammar errors. We thank reviewer 1 for noticing the spelling errors in the MS***

4- In the discussion section, the first paragraph should mention the main findings of your study. Please add your most valuable and novel findings of your paper to the first paragraph.

**RESPONSE: *We appreciate the reviewer's comment. We have rewritten the first paragraph of the Discussion section. Please see lines 275-279 in Tracked version.***

5- Add new headings named "Strengths and limitations" and "Conclusions" to the discussion section. Add the strengths of your study.

**RESPONSE: *We have added* "Limitations and strengths" and "Conclusions" *sections in the MS. Please see lines 347-366 in Tracked version.***

Reviewer #2: I consider that the study is, simple, and clear but very interesting. The conclusions are in accordance with the results obtained. To enhance the presentation of the manuscript, I have contributed with some corrections of form with the correct names of the mice strains used and the forms of expression of some numbers or decimal or signs in papers.
Also, I have modified some mistakes and the way in which we have to call the patients

**RESPONSE: *We appreciate all the comments recommended by the reviewer #2 to improve the manuscript. We have revised cautiously the MS, replacing words as” Balb/c” and “chagasic”. We have added a space between the last number and the % sign in all the MS. Italics words have been added appropriately.***
